# The association between malaria and non-typhoid Salmonella bacteraemia in children in sub-Saharan Africa: a literature review

**DOI:** 10.1186/1475-2875-13-400

**Published:** 2014-10-13

**Authors:** Ebako Ndip Takem, Anna Roca, Aubrey Cunnington

**Affiliations:** Ministry of Health, BP 281, Buea, Cameroon; Medical Research Council Unit, Atlantic Road Fajara, PO Box 273, Banjul, The Gambia; Section of Paediatrics, Imperial College London, Norfolk Place, London, W2 1PG UK

**Keywords:** Malaria, Non-typhoid Salmonella, Children, Epidemiology, Pathogenesis

## Abstract

*Plasmodium falciparum* malaria and non-typhoid Salmonella (NTS) bacteraemia are both major causes of morbidity and mortality in children in sub-Saharan Africa. Co-infections are expected to occur because of their overlapping geographical distribution, but accumulating evidence indicates that malaria is a risk factor for NTS bacteraemia. A literature review was undertaken to provide an overview of the evidence available for this association, the epidemiology of malaria-NTS co-infection (including the highest risk groups), the underlying mechanisms, and the clinical consequences of this association, in children in sub-Saharan Africa. The burden of malaria-NTS co-infection is highest in young children (especially those less than three years old). Malaria is one of the risk factors for NTS bacteraemia in children, and the risk is higher with severe malaria, especially severe malarial anaemia. There is insufficient evidence to determine whether asymptomatic parasitaemia is a risk factor for NTS bacteraemia. Many mechanisms have been proposed to explain how malaria causes susceptibility to NTS, ranging from macrophage dysfunction to increased gut permeability, but the most consistent evidence is that malarial haemolysis creates conditions which favour bacterial growth, by increasing iron availability and by impairing neutrophil function. Few discriminatory clinical features have been described for those with malaria and NTS co-infection, except for a higher risk of anaemia compared to those with either infection alone. Children with malaria and NTS bacteraemia co-infection have higher case fatality rates compared to those with malaria alone, and similar to those with bacteraemia alone. Antimicrobial resistance is becoming widespread in invasive NTS serotypes, making empirical treatment problematic, and increasing the need for prevention measures. Observational studies indicate that interventions to reduce malaria transmission might also have a substantial impact on decreasing the incidence of NTS bacteraemia.

## Background

There are thousands of serotypes of Salmonella, including those grouped as *Salmonella enterica* subspecies enterica, which can cause disease in humans, and are normally dichotomized into those causing typhoid fever (i.e., *S. enterica* subsp. enterica serotype Typhi, and Paratyphi), and non-typhoid salmonella (NTS) serotypes which include Enteritidis and Typhimurium [1, 2]. Infections due to NTS in humans are an important cause of enteric infections and invasive disease [3]. Enteric infections can lead to invasive disease which includes bloodstream infection (bacteraemia) and/or focal disease (for example, pneumonia, meningitis and osteomyelitis). NTS bacteraemia is a major cause of morbidity and mortality in African children and immunocompromised adults [3–8]. Invasive NTS disease has particularly been associated with the emergence of the ST313 strain of S. Typhimurium in sub-Saharan Africa [9, 10]. In this review, NTS bacteraemia is considered synonymous with symptomatic bloodstream infection since the diagnosis is usually made in symptomatic individuals admitted in health facilities.

Malaria is caused by five species of *Plasmodium* parasites that are known to affect humans and include *Plasmodium falciparum, Plasmodium ovale, Plasmodium malariae, Plasmodium vivax*, *and Plasmodium knowlesi* [11]. The greatest burden of disease is caused by *P. falciparum* and is also borne by children in sub-Saharan Africa [11].

Paediatric NTS bacteraemia and malaria overlap in terms of geographical distribution, age groups at greatest risk and seasonality of both diseases in the tropics. The effect of malaria on susceptibility to NTS bacteraemia was suspected in British Guiana in the early part of the last century [12], but relatively few epidemiological studies [13–17], involving human populations were subsequently done to better quantify or confirm the association. Most of the epidemiological evidence in humans is based on these studies, and on studies showing parallel decreases in incidence of malaria and NTS bacteraemia in the same geographical area over time [18, 19]. Only one of these published reports included suitable community controls [14]. Numerous hospital-based studies have shown higher prevalence of malaria parasites among children hospitalized with NTS compared to other bacteraemia/other admitted children [6, 13, 15, 16, 20, 21], or a higher prevalence of NTS bacteraemia among those with malaria parasites compared to aparasitaemic children [4, 22, 23], but their interpretation is very challenging, as they could be subject to selection bias. Nevertheless, a number of recent studies have improved current understanding of underlying mechanisms, and show convincingly that malaria really does increase the risk of NTS bacteraemia. This review focuses on the available literature on this association in children in sub-Saharan Africa and the underlying biological mechanisms.

## Search strategy

A literature search was undertaken in the PubMed database using the search terms “Malaria”, “Salmonella” and “Africa”. Articles that reported the occurrence of malaria and NTS in children, were selected. Additional articles were extracted from the references lists of the full publications and from the authors’ personal collections.

## Burden

NTS are one of the leading causes of bacteraemia in sub-Saharan African countries [4–6, 13, 15, 24–28]. They are among the two most common causes of bacteraemia in children [4, 24–27], and also a major cause of bacteraemia in immunocompromised adults with HIV [5, 28]. The greatest burden of invasive NTS disease is in children, especially those under three years of age [26, 27]. Incidence rates of NTS disease among hospitalized children range from 88–300 cases per 100,000 in some African settings [25–27], but the actual burden may be higher than this since a great proportion of children may not be seen in health facilities, and high quality microbiologic diagnostics may not always be available. In addition, estimation of invasive NTS incidence based on routine blood cultures will underestimate the true incidence due to the low sensitivity of this technique [25, 29]. The proportion of all pathogenic isolates that are NTS, obtained from health facility-based studies, is variable and ranges from 2-77% in children [4, 6, 7, 24, 26, 27, 30, 31]. Of the NTS serotypes, S.Typhimurium and S.Enteritidis usually account for the majority (>80%) of the NTS isolates in blood [5, 7, 15, 26, 30, 32–37]. The great variation in the incidence of NTS in the different studies probably indicates differences in enrolment criteria between the studies; for example, age differences, in children and clinical characteristics, study setting (Table 1), underlying host factors such as differences in haemoglobinopathies, and malnutrition. The burden of NTS may be linked to the invasive potential of the dominant NTS serotypes [9, 10].

**Table 1:** **Studies reporting malaria and NTS bacteraemia in children**

Study site	n	Age	Study population	NTS bacteraemia diagnosis	Malaria diagnosis	Major findings and comments
Burkina Faso (rural) [68]	711	<15 y	All admitted children with measured fever or clinical signs of severe illness	BC	M, RDT	RDT positivity rate was higher in those with NTS bacteraemia (81%) compared to those with other bacterial infections (31%) (p <0.001)
Tanzania (rural + urban) [16]	3,639 + 457	2 m-13 y	Admitted children with measured fever or history of fever	BC	M, RDT	Children with recent malaria had higher rates of NTS bacteraemia compared to those without recent malaria (adjusted OR =4.13(95% CI = 2.66-6.44)
DRC (mainly rural) [69]	3,311	<=14 y and adults	Signs suggestive of bacteraemia or focal signs	BC	M and/or RDT	Majority of children (82%) with Salmonella had falciparum malaria infection, NTS not seasonal, comparison group not mentioned
Kenya (rural + urban) [5]	5,716	-	Children with fever, severe respiratory illness, admitted patients	BC	M	Evidence of correlation between positive malaria cases and NTS bacteraemia, no clear seasonal pattern, no comparison group
Kenya (rural) [14]	292	3 m-13 y	Cases: admitted children whose BC grew pathogenic bacteria	BC	M or RDT	Those with haemozoin in blood leucocytes were more likely to have NTS bacteraemia compared to those without haemozoin OR 16.5 (95% CI = 3.44-79.3)
			Controls: healthy children individually matched to cases on age, sex and residential location			
Tanzania (rural) [18]	6,836	2 m-14 y	History of fever, clinical signs of severe malaria, fever surveillance	BC	M and RDT	Evidence of reduction in NTS bacteraemia associated with severe malaria reduction
Kenya (rural) [58]	585	1-36 m	Children with malaria aged 1–36 m	BC	M	NTS was the most common isolate in children with malaria, comparison group not mentioned
DRC (rural) [20]	1,528	-	Febrile children admitted, hypothermia, other clinical signs	BC	M	40% of NTS bacteraemia had malaria co-infection compared to 1% for typhoid bacteraemia, no seasonality of NTS
Tanzania (rural) [6]	1,502	2 m-14 y	Fever + signs of severity	BC	M or RDT	73% with NTS infection had malaria compared to 21% for those with typhoid fever (p < 0.01) and compared to 40% for other bacteraemia (p < 0.01) - association more for recent than current malaria
Ghana (rural) [64]	948, 1,032 cultures	2 m-5 y	Children 2 m-5 y admitted	BC	-	24% of children with NTS bacteraemia had malaria infection compared to 18% for other bacteraemia (*S. pneumoniae*), no significance test mentioned
Tanzania (rural) [4]	3,639	2 m-12 y	Fever, non-infectious cause of fever excluded	BC	M, RDT	52% NTS in slide positive compared to 45% in slide negative, no significance test mentioned
The Gambia (rural + urban) [19]	-	-	-	BC	-	NTS reduction associated with malaria reduction
Kenya (rural) [22]	3,068^a^	-	Children with clinical suspicion of severe malaria and culture results available	BC	M	NTS more in parasitaemic children compared to non-parasitaemic children (p = 0.05)
Mozambique (rural) [104]	1,780	<5 y	Children <5 y with severe malaria	BC	M	NTS among frequent bacteria in patients with severe malaria but no evidence of association
Mozambique (rural) [26]	23,686	<15 y	Children <15 y admitted	BC	M	About 44% of bacteraemic patients had malaria co-infection. No 7comparison with control and no mention of NTS specifically
Nigeria (urban) [118]	235	0-45 m	Children with fever with or without other symptoms	BC	M	Co-infection with *S. enteritidis* and malaria present, no mention of control group
The Gambia (urban) [41]	871	2 m-80 y	Clinically ill patients	BC	M	NTS 20% in slide positive compared to 57% in slide negative but not statistically significant, few cases of NTS
Tanzania (urban) [65]	1,787	0-7 y	Clinical suspicion of systemic infection	BC	M	No evidence of association between malaria and NTS
Malawi (urban) [24]	1,388	≥6 m	Children with severe malaria and BC results	BC	M	NTS bacteraemia higher in those with severe malaria anaemia (7.6%) compared to other severe malaria entities [CM + SMA] (4.7%) compared to CM (3.0%) p <0.0001]
Kenya (urban) [66]	332	4 w-84 m	NTS bacteraemia or gastroenteritis	BC	M	More than half of malaria confirmed children had NTS, no seasonal pattern. Proportion in control group not mentioned
The Gambia (rural) [27]	330	2-29 m	Ill children admitted	BC	M or RDT	No difference in proportion of malaria infection between those with NTS infections compared to other infections
Kenya (rural) [15]	166	<13 y	Children with Salmonella bacteraemia	BC	M or RDT	More NTS in rainy season; recent malaria (RDT positive) but not current malaria was a risk factor for NTS bacteraemia compared to non-bacteraemic patients (OR = 1.8, 95% CI 1.0-3.1)
Kenya (rural) [43]	2,830	>3 y	Children admitted for malaria (parasite positive) and for other illnesses (parasite negative)	BC	M	Salmonella spp. bacteraemia more common in those parasite positive. No specific mention of NTS bacteraemia
DRC (rural) [23]	779	1 m-15 y 8 m	Children with and without fever	-	M	A positive blood smear associated with bacteraemia (including NTS). There was enough evidence that 25% of malaria positive had bacteraemia compared to 14% for malaria negative
Malawi (urban) [13]	2,123	<1-15 y	Children with clinical suspicion bacteraemia (febrile) and low level parasitaemia or after anti-malarial	BC	M	Children with NTS bacteraemia more likely to have parasitaemia compared to other bacteraemia (RR 2.4, 95% CI 1.46-3.96), NTS increase in rainy season
Malawi (urban) [21]	299	0-14 y	Sick children with NTS bacteraemia, focal sepsis excluded	BC	M	NTS increase in rainy season, coincides with malaria, NTS associated with severe anaemia, malaria parasitaemia compared to other causes of bacteraemia
Kenya (rural) [63]	783	-	Children with severe malaria	BC	M	6 out of 540 children with severe malaria (and BC results available) had NTS, bacteraemia common in children with severe malaria
DRC (rural) [29]	120	1-15 y	Clinically ill in wards and outpatient	BC	M	Concurrent malaria parasitaemia and bacteraemia in 25% of cases
The Gambia (rural) [70]	2,898	<5 y	Clinical signs of pneumonia, meningitis, septicaemia	BC	M	Salmonella bacteraemia increased during rainy season, those with malaria pigment more likely to be found in those with Salmonella infections compared to other infections (RR = 4.05, 95% 1.15-14.42), comparison not done specifically for NTS
Nigeria (rural + urban) [67]	56	<5 y	Case series with positive BC, referred	BC	M	Increase in cases of paratyphoid fever during rainy season
The Gambia (urban) [17]	247		Clinically ill children with positive blood culture	BC	M	Patients with NTS bacteraemia had higher prevalence of malaria parasitaemia compared to other bacteraemic patients (X^2^ = 9, p < 0.01)

All health facility-based studies in Table 1.

BC = blood culture, M = microscopy, RDT = rapid diagnostic test, RR = relative risk, CM + SMA = cerebral malaria and severe malarial anaemia.

^a^These children were compared to 592 controls from the community.

In 2013, there were 104 countries and territories around the world in which malaria was considered endemic [38]. An estimated 3.4 billion people around the world are currently at risk of malaria, and in 2012, more than 80% of cases and 90% of deaths were in WHO African region, with pregnant women and children aged under five years being at highest risk [38]. Malaria due to *P. falciparum* is the most common form in sub-Saharan Africa and is responsible for the vast majority of severe disease. Other *Plasmodium* species are less common, and their association with NTS has not been systematically studied. Both pathophysiological and/or epidemiological factors may account for the absence of any reported association, although there are a few reports of bacteraemia and *P. vivax* co-infection outside sub-Saharan Africa [39].

**Figure 1:**
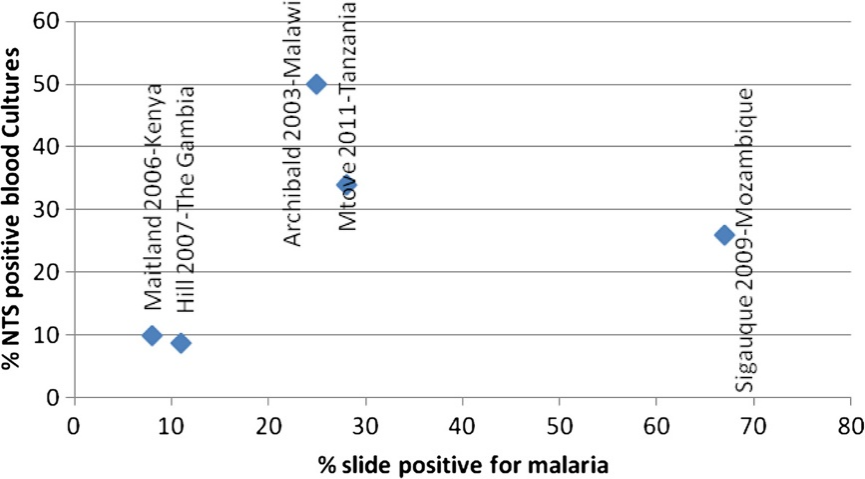
**Rates of malaria and NTS bacteraemia in some selected settings of low and high malaria burden** ^**a**^ **.** ^a^Studies included in which the slide positivity rate and the proportion of all pathogenic isolates that are NTS were both reported. x-axis corresponds to the parasite positivity rate ie number positive for malaria/total number of slides read. y-axis corresponds to the proportion of all pathogenic isolates that are NTS ie proportion of positive blood cultures that were positive for NTS.

Although a decrease in malaria burden has been recorded in some African countries in the past decade, there are still a large number of countries where no reduction has been achieved [11]. In many countries where a substantial decline in malaria has been described, a corresponding reduction in the rate of NTS has also been documented [18, 19, 40, 41]. While in countries with sustained high malaria burden, the rates of NTS remain a major public health problem [4, 5, 15, 25, 42–44]. Figure 1 shows the rates of malaria and NTS in studies conducted in settings with different malaria burden.

There are relatively few published studies on NTS asymptomatic faecal carriage and seasonality in sub-Saharan Africa. A study conducted in The Gambia showed that NTS faecal carriage remained the same throughout the year, as opposed to NTS bacteraemia cases that were seasonal [17], with the peak coinciding with the malaria season. These taken together, may suggest that, malaria increases the risk of NTS bacteraemia in those who are already infected (carriers) rather than increasing the risk of carriage of NTS, but further studies are needed to confirm this hypothesis.

## Source and transmission of NTS

In most countries in sub-Saharan Africa, the majority of NTS infections are community acquired and believed to be related to contaminated water, food, livestock, and poultry [45, 46], although evidence for the transmission from animals to humans is mostly indirect [47–49]. There are few data on human-to-human transmission, but there is emerging evidence it may occur in children [34], and nosocomial infections have been reported in hospital wards in South Africa [50]. NTS bacteraemia follows entry through the gut [51], which might explain why NTS infections are strongly associated with malnutrition, when the gut mucosal barrier is impaired [52]. This might also explain why NTS disease increases during the rainy season, when the gut mucosal barrier is impaired by malaria parasite sequestration [53, 54]. Nevertheless, some other factor, which increases in the rainy season, may be responsible for increase in susceptibility, as seasonality in NTS bacteraemia has also been observed in non-malarious areas [55, 56].

## Risk factors for NTS bacteraemia

Generally, the risk of NTS bacteraemia is higher in children, especially those under three years of age, compared to adults [26, 27]. In addition, the risk of NTS bacteraemia tends to be higher in rural compared to urban settings [5]. In children, the main risk factors for NTS bacteraemia are younger age [5, 6, 34], anaemia (independent of malaria) [13, 15, 21, 57], malnutrition [15, 26, 58, 59] especially severe malnutrition, sickle-cell disease [60, 61], HIV [15, 25], and malaria [4–6, 13, 15, 17, 27]. The interactions between these risk factors (for example malnutrition, sickle-cell disease, HIV and malaria) appear to be complex, and beyond the scope of this literature review.

### Malaria

A number of studies have reported malaria and NTS bacteraemia in the same populations of children in sub-Saharan Africa [4–6, 13–17, 20, 21, 23, 26, 27, 62–66], although only a few of them [14–17], were specifically aimed to evaluate the effect of malaria on the risk of NTS bacteraemia (Table 1). In the majority of these studies that evaluated the effect of malaria on NTS bacteraemia [15–17], health facility controls were used, and could be subject to selection bias. Thus, comparisons of prevalence of parasitaemia between NTS cases with other predefined hospitalized patients may over or underestimate the effect of malaria on risk of NTS bacteraemia. In malaria endemic areas, the rate of NTS bacteraemia increases during the high transmission/rainy season, and it is believed that malaria probably contributes to the seasonality of NTS [13, 15, 17, 19, 21, 30, 62, 67]. A more precise description of the association between malaria and NTS is also made difficult by variation in how different studies have defined malaria exposure. Current malaria is usually defined as asexual stages of *P. falciparum* on a blood film together with compatible clinical symptoms, whilst recent malaria has been variably defined based on: the presence of malaria pigment (haemozoin) in blood leucocytes; the presence of gametocytes but not asexual parasites on the blood film; or a positive rapid diagnostic test (RDT) in the presence of a negative blood film. However, many studies have defined malaria exposure in a way that may represent a composite of these groups. Surprisingly few studies have explicitly reported the independent associations of current or recent malaria with NTS bacteraemia.

Only two studies that explicitly reported the association of current malaria with NTS bacteraemia in children were identified [13, 17]. A study in urban Malawi showed that those with current malaria infection (parasitaemia) were more likely to have NTS bacteraemia (about two times more) compared to those without malaria parasitaemia (Table 1) (RR =2.4, 95% CI 1.46-3.96) [13]. In this health facility-based study with a cross-sectional design, the controls were admitted children who did not have positive blood culture for NTS. Another study that evaluated the effect of current malaria infection in an urban area of The Gambia showed that patients with NTS bacteraemia had higher prevalence of malaria parasitaemia compared to other bacteraemic patients (X^2^ = 9, p < 0.01) [17].

Similarly, four studies that explicitly reported an association with recent malaria were identified [6, 14–16]. In a study conducted in Tanzania, the proportion of those with a positive RDT and a negative blood film was higher in those with NTS bacteraemia (20/45, 44%) compared to 13/97 (13.4%) in those with other bacteraemia [6]. In a study conducted in rural Kenya, children with NTS bacteraemia in a health facility were compared to community controls in a matched case–control study [14]. Those with haemozoin (malaria pigment) visible on blood films were close to 17 times more likely to develop NTS bacteraemia compared to those without visible malaria pigment. In addition, HbAS genotype was associated with protection against bacteraemia, which was mediated by the known protection of HbAS against malaria. In the same report, which included a longitudinal study, there was a reduction in the incidence of NTS bacteraemia associated with a reduction in malaria infection [14]. Children with detectable *P. falciparum* histidine rich protein 2 (*Pf*HRP2) and negative blood film were about two to four times more likely to have NTS bacteraemia compared to those without any evidence of recent malaria [15, 16]. A health facility-based study in Tanzania showed that children with detectable *Pf*HRP2 and negative blood film were more likely to have NTS bacteraemia compared to those without (adjusted OR =4.13, 95% CI 2.66-6.44) [16]. Another study conducted in rural Kenya showed that children with detectable *Pf*HRP2 and negative blood film had a higher risk of NTS bacteraemia compared to those without (OR = 1.8, 95% CI 1.0-3.1) [15]. It should be noted that this study also compared children with NTS bacteraemia to those with other bacteraemia who were admitted in a health facility. A recent study in health facilities in rural Burkina Faso reported that the proportion of children with RDT-positive malaria was higher in those with NTS bacteraemia (81%) compared to those with other bacterial infections (31%) (p < 0.001) [68], although it was unclear whether these RDT-positive participants had a negative blood film. In studies that explicitly evaluated both current and recent malaria, the association with NTS bacteraemia was observed more [6], or solely [15], for recent malaria. This may indicate that the mechanism by which malaria causes susceptibility to NTS lags behind the acute infection, or it may simply reflect the time at risk, since a current malaria episode is limited relatively quickly by seeking treatment, whereas malaria antigens and haemozoin may persist for weeks to months, respectively.

Despite some limitations, the aforementioned studies have provided accumulating body of evidence that has been consistent with an association between symptomatic/recent malaria and NTS bacteraemia in sub-Saharan African children. Whilst there is evidence for the association between symptomatic malaria and NTS bacteraemia, few data on the association between asymptomatic malaria infections and NTS bacteraemia, were found. There is some evidence that among children with severe malaria, those with severe malarial anaemia have a higher risk of NTS bacteraemia [24], suggesting that this might be due to haemolysis. It should be noted that this is based on relatively few studies. There is lack of data on the risk of NTS in those with severe malaria compared to those with mild malaria.

Some studies have reported an association between malaria parasitaemia and any Salmonella bacteraemia (Typhoidal or NTS) [23, 69, 70]. One of the studies, which involved health facilities in a rural area in The Gambia, showed that those with haemozoin in their leucocytes were more likely to have invasive Salmonella infections compared to those without haemozoin in their leucocytes (RR = 4.05, 95% CI 1.15-14.42) [70].

## Mechanisms of susceptibility to NTS bacteraemia

### Host defence against invasive NTS

Studying the pathogenesis of NTS bacteraemia in humans is difficult, but findings from animal models have suggested a plausible sequence of events from the arrival of NTS in the intestine to invasion, bacteraemia and dissemination to other organs. Invasive disease begins with invasion of epithelial and M cells of the intestine, facilitated by the proteins of the Salmonella Pathogenicity Island 1, Type Three Secretion System (T3SS1), which induce internalization of bacteria by massive rearrangement of the host cell membrane [51]. Bacteria then invade, or are phagocytosed by, macrophages and dendritic cells in the submucosa and mucosa-associated lymphoid tissues, where another set of proteins of the T3SS2 are activated to promote intracellular survival and replication [71, 72]. Bacteria begin to disseminate through the lymphatic system draining the intestine, and may also pass directly into the blood stream. These bacteria may be carried intracellularly in migrating cells, or become extracellular after pyroptosis (inflammatory cell death) of infected cells. Ultimately they reach the phagocytic cells of the reticuloendothelial system (liver, spleen and bone marrow) and establish new foci of infection from which they can disseminate [71]. Early containment of infection in the submucosa of the intestine is dependent on activation of the innate immune response and early production of interleukins (IL) IL-1β, IL-18, IL-23 and Tumour Necrosis Factor (TNF). This early response solicits local chemokine production, influx of monocytes and neutrophils and interferon-γ production by T-cells, which is critical for control of intracellular bacteria [73, 74]. Killing of bacteria at this early stage is particularly dependent on the oxidative burst in phagocytic cells [75], and it is likely that antibodies against NTS are also important in limiting this initial infection and dissemination of bacteria [74]. Cell-mediated immunity is likely to have a major contribution as infection proceeds, primarily by a Th1 response (most prominently IFN-γ) facilitating intracellular killing mechanisms [74, 76]. One important caveat, is that the commonly used model system, S. Typhimurium infection in the mouse, has been primarily used as a model of Typhoid [71], and may not represent all of the features of the most invasive ST313 strain in African children. However, in broad terms the conclusions about mechanisms of host defence against NTS are supported by observations of susceptibility to NTS in humans with a variety of primary and acquired immunodeficiencies including: innate immune defects affecting the IL-12/IL-23/Interferon-γ axis [77]; chronic granulomatous disease (in which phagocytic cells cannot mount an effective oxidative burst) [78]; MHC class II deficiency; treatment with TNF neutralizing antibodies (for inflammatory diseases such as Crohn’s disease and rheumatoid arthritis) [74]; and HIV (in which impaired mucosal integrity, CD4 lymphopaenia, immune dysregulation and abnormal antibody production are all implicated [1, 79, 80].

### Modulation of host defence against NTS by malaria

Numerous mechanisms have been proposed to explain the susceptibility to NTS induced by malaria, including impairments of: mucosal barrier function; macrophage function; neutrophil function; and, antibody production. *Plasmodium falciparum* is particularly distinguished by its propensity for cyto-adherence of infected red blood cells to vascular endothelium, a phenomenon known as sequestration [53]. This is believed to permit the generation of very high parasite loads in the human body, by avoiding clearance in the spleen, and also to contribute to pathology by obstruction of flow in small blood vessels. Extensive sequestration of parasitized red blood cells in the microvasculature of the intestine is a common finding in *post-mortem* studies of fatal *P. falciparum* malaria [54]. Impaired microvasculature blood flow might directly lead to impaired mucosal barrier function, and reduce resistance to invasion by NTS [22, 81]. In addition, L-arginine deficiency which is induced by malaria, has been shown to increase intestinal permeability and bacterial translocation, and may exacerbate this effect [82].

Macrophage dysfunction has been one of the most attractive explanations for the susceptibility to NTS in malaria [17], because macrophages are one of the most important cells harbouring NTS in standard models of infection. A variety of reasons for macrophage dysfunction have been proposed including haemozoin ingestion [83], erythro‒/haemo‒phagocytosis [84] or reduced production of cytokines such as IL‒12 (which is necessary to facilitate the killing of intracellular NTS) [85]. Erythropoietin has recently been described to have a role in the impairment of resistance to NTS in mice [86]. Erythropoietin is the main regulator of erythropoiesis in bone marrow, but its receptors are also expressed on other cell types and appear, amongst other effects, to regulate the inflammatory function of macrophages [87]. Consistent with this, erythropoietin levels are generally elevated in severe malarial anaemia, the major risk factor for NTS, as would be expected as part of the homeostatic response to severe anaemia [88]. However, an important caveat is that it is unclear to what extent the standard model of pathogenesis applies during malaria-NTS co-infection, and particularly whether macrophages are the main cell type harbouring NTS. As detailed below, neutrophils appear to provide a new niche for bacterial replication during co-infection and may be more important than macrophages in this respect.

Relatively little attention has focused on the role of humoral immunity and the effect of malaria on the natural acquisition of antibodies against NTS. Antibodies play a clear role in protection against NTS bacteraemia, and are usually acquired during the second year of life in African children [76]. Malaria can suppress the generation of heterologous antibody responses, and recurrent episodes of malaria during early childhood might suppress natural acquisition of antibodies to NTS, just as they suppress antibody responses to Salmonella capsular polysaccharide vaccine [89]. Lack of antibody may also reduce the efficiency of neutrophil-mediated oxidative killing of NTS [90]. Disruption of splenic architecture during malaria is one likely mechanism for humoral immune dysfunction [89], but may also directly impair the splenic clearance of NTS [91].

Despite these many possibilities, one of the most consistent and compelling observations to explain the relationship between malaria and susceptibility to NTS is the causal role of haemolysis. Apart from malaria, several other haemolytic diseases are associated with susceptibility to NTS, namely sickle cell disease and Carrion’s disease (acute Bartonellosis) [92, 93]. Consistent with this, any cause of haemolysis (including malaria infection) will induce susceptibility to NTS in mice [94–96]. Interestingly, in this model, bacteraemia is a prominent feature, as seen in human NTS-malaria co-infections, and replicating bacteria are particularly found concentrated inside neutrophils in the blood [97]. The mechanism underlying this susceptibility was shown to be an indirect consequence of host mechanisms that promote survival in malaria. Haemolysis can be extremely damaging if its extent overwhelms host mechanisms to prevent liberation of free haem from haemoglobin [98, 99]. Haem oxygenase-1 (HO-1) is the inducible haem-degrading enzyme, which protects against haem-mediated toxicity and is essential for survival in malaria in mice. In addition to protecting against haem toxicity, HO-1 and the haem-degradation products, carbon monoxide and biliverdin, all have diverse immunomodulatory and cytoprotective effects. Amongst these effects, HO-1 induction has been associated with suppression of oxidative burst activity, which could benefit the host by preventing oxidative cytotoxicity in severe malaria, but might be disadvantageous when combating NTS. Indeed, malaria was shown to cause HO-1 dependent impairment of the neutrophil oxidative burst in mice, and this was apparent during neutrophil maturation in the bone marrow [97]. However, susceptibility was also dependent on haem, possibly as a source of iron for the siderophilic NTS, and could be reversed by competitive inhibition of HO-1. A similar defect in neutrophil oxidative burst activity was subsequently demonstrated in Gambian children with *P. falciparum* malaria, which correlated with the extent of haemolysis and HO-1 induction, and interestingly persisted for weeks beyond the clearance of acute malaria infection [100]. No studies have yet been performed to investigate prospectively whether the extent of impairment of neutrophil oxidative burst function associated with malaria correlates with risk of subsequent invasive NTS infection.

## Clinical presentation and diagnosis

### Clinical presentation

There is much literature on the clinical presentation of bacteraemia but little specifically on NTS and malaria co-infection, which is the subject of this review. Generally, the signs of NTS bacteraemia are clinically difficult to distinguish from other causes of bacteraemia in children [21, 101], characterised by non-focal sepsis. Nevertheless, data on the clinical presentation of NTS bacteraemia in children indicate that apart from the usual signs of sepsis, the following may be prominent: anaemia, splenomegaly, respiratory, and gastrointestinal signs [6, 13, 15, 21, 101]. Only one study that compared clinical features in children with co-infection with those in children with malaria alone, was identified [6]. Anaemia, particularly severe anaemia, tends to occur more frequently in those with NTS bacteraemia compared to those with malaria, or those without bacteraemia, or those with other pathogenic bacteria [6, 13, 15, 21]. A recent review of 25 studies across 11 countries in sub-Saharan Africa confirmed that a higher proportion of children with severe malaria anaemia had NTS bacteraemia [102]. Splenomegaly is more frequent in children with NTS bacteraemia compared to non-bacteraemic or other causes of bacteraemia [13, 15]. Other clinical features such as fever, jaundice, hypoglycaemia, malnutrition, and diarrhoea have also been reported to be more frequent in NTS bacteraemia compared to those with malaria only or other causes of bacteraemia [6, 27]. Respiratory features have been described both clinically and radiologically in conjunction with NTS bacteraemia, and include signs of lower respiratory tract infection and effusion or consolidation [15, 27], however whether these are caused by NTS itself or another co-infecting pathogen remains to be determined. White blood cell counts appear to be higher in children with NTS compared to others [27].

### Diagnosis

Blood culture is the most widely used method for diagnosis of NTS bacteraemia in most studies, although PCR methods have been developed and may prove useful in future studies [103]. The sensitivity of blood culture for NTS in sub-Saharan African children was not found in the published literature, but a study in HIV-infected Malawian adults suggested that the viable bacterial load in blood is low (about 1 CFU/mL), and so small volume blood culture samples that are often taken in children may have limited sensitivity [80]. There are no similar data in humans with malaria and NTS co-infection, but in mice with co-infection, bacteria were found to be particularly concentrated in blood [97].

## Complications

Data available on mortality show that the case fatality rate for children with NTS bacteraemia ranges from 12-24% [21, 101]. A recent review published in 2012 showed that the case fatality rates in African adults and children, with invasive NTS bacteraemia, ranged from 20-25% [1]. Another more recent review published in 2014 showed that mortality among those with malaria and concomitant invasive bacterial infections was higher, 24.1% (95% CI 18.86-29.36) compared to those with malaria infection alone, 10.2% (95% CI 9.33-10.98), and this higher mortality was similar specifically for malaria and concomitant NTS infections [102]. In general, mortality is higher in those with bacteraemia (including NTS), compared to those without bacteraemia, irrespective of malaria infection [4, 5, 25, 42, 58, 63]. NTS bacteraemia was associated with higher risk of death in children in Tanzania [6] compared to those with malaria. Those with bacteraemia and malaria co-infection have a higher case fatality compared to those with malaria infection only [43, 104]. Thus the presence of bacteraemia increases the risk of death in children with malaria. The risk of death in those with bacteraemia and malaria co-infection is similar to those with bacteraemia alone [13, 21], i.e., the presence of malaria does not seem to increase the case-fatality rate in bacteraemic patients. Among bacteraemic patients, if the aetiology of bacteraemia is considered separately, those with NTS have a lower mortality compared to those with other causes of bacteraemia, like other enteric Gram-negative rods and *Streptococcus pneumoniae* [13]. Anaemia, lower age, and HIV are associated with higher risk of mortality in those with NTS [21, 105]. Prior antimicrobial therapy might influence the case-fatality rates associated with bacteraemia [101].

Data on mortality among those with severe malaria and NTS are sparse. If the different severe malaria syndromes are considered in those with co-infection, then those with severe malaria anaemia and those with cerebral malaria have a higher mortality compared to non-bacteraemic and compared to other severe malaria syndromes [24, 104].

## Prevention and management

### Prevention

The most pragmatic way to prevent malaria and NTS co-infection may be to prevent malaria. There is abundant evidence that conventional strategies to reduce malaria transmission result in reductions in both malarial and non-malarial morbidity and mortality [106–108]. Furthermore, reductions in malaria transmission (at least partly due to control measures), in at least three distinct epidemiological settings, have been associated with reductions in the incidence of NTS bacteraemia [14, 18, 19]. However, most of this evidence is from observational studies, and to date there have been no controlled trials to confirm that reducing malaria transmission is an effective method to reduce NTS bacteraemia.

To prevent NTS bacteraemia in children who already have malaria, targeted antibiotic prophylaxis for children at high risk of subsequent NTS infection, for example those with severe anaemia, may represent an effective strategy. Another intriguing possibility is inhibition of haem oxygenase activity with tin protoporphyrin [97, 109], and which might be administered after treatment for malaria in order to reverse haem oxygenase-1-mediated neutrophil dysfunction. Neither of these has yet been evaluated in a clinical trial, but they may not be difficult to combine with existing malaria treatment strategies.

#### Vaccination

The most advanced candidate vaccine for malaria, the RTS,S vaccine, may play an important role in reducing malaria episodes, and consequently bolster the effect of conventional control measures [110]. Even blood stage vaccines, which might reduce parasitaemia without preventing episodes of infection, might have a substantial effect on reducing the risk of NTS bacteraemia, by limiting the amount of haemolysis. There has been some recent interest in using S. Typhimurium-*Plasmodium* fusion proteins, with Salmonella flagellin improving the immunogenicity of the parasite antigen [111]. Since Salmonella flagellin can also induce protective immune responses against S. Typhimurium [112], there is the intriguing possibility that the combination of antigens from both pathogens might result in a vaccine that is efficacious against each of them. There is currently no licensed vaccine to prevent NTS in humans, and most candidates are at very early stages of development. Outer membrane protein antigens, flagellin, and the lipopolysaccharide O antigen are promising targets [113], and have been assessed in both glycoconjugate and live-attenuated vaccination strategies [114, 115]. There is a pressing need to develop an efficacious vaccine against invasive NTS disease, but to have maximal impact, it will need to be safe and effective in both the youngest children and individuals with HIV.

### Management

Prior to the availability of antibiotics, it was reported that some adults with malaria-NTS co-infection were cured with quinine alone, implying that treatment of malaria was sufficient to allow the host response to clear the NTS bacteraemia [12]. Such a management strategy would no longer be acceptable, and combined anti-malarial and antibiotic treatment would be essential. Malaria can usually be diagnosed rapidly (by blood film or RDT), but diagnosis of NTS bacteraemia requires at least 24–48 hours for growth in blood cultures (which are unavailable in many resource-poor settings), and so a major problem is deciding which patients with malaria should receive empirical antibiotic therapy [4]. Recent work by Hendrikssen *et al.* indicates that a quantitative measure of PfHRP2 – which is already the basis of many RDTs – might help to identify those most at risk of having co-incident bacteraemia [116]. Children appearing to have severe malaria, with either very high or very low levels of PfHRP2 had the highest risk of bacteraemia, and might be targeted for empirical antibiotics. Arising from this is a second problem – what empirical antibiotic is most appropriate? Although NTS is often reported as the most common bacterial pathogen causing co-infections in children with malaria, other bacteria also need to be considered, including: *Streptococcus pneumoniae*, *Escherichia coli*, *Haemophilus influenzae*, *Acinetobacter* species and *Pseudomonas aeruginosa* [22].

However, antimicrobial resistance among NTS isolates is an increasing problem worldwide, particularly with spread of the ST313 clone of S. Typhimurium. Multidrug resistance to commonly used antimicrobials, such as ampicillin, chloramphenicol and cotrimoxazole, has been reported to be common in a variety of African settings [26, 27, 30, 34, 117, 118]. Selections of empirical antibiotic treatment should be made based on local epidemiology and resistance data wherever possible, but it is clear that many of the older (cheaper) antimicrobials may no longer be adequate, and treatment with a third generation cephalosporin, or combination therapy including ciprofloxacin, may be necessary to give a broad enough coverage and reasonable chance of cure in children with malaria and suspected bacterial co-infection [1, 4].

## Conclusions

Accumulating epidemiological and preclinical evidence supports the causal association between malaria and NTS bacteraemia. However, the clinical characteristics and consequences of malaria and NTS co-infection are not well defined, although mortality associated with co-infection appears higher than that associated with malaria alone. Future observational studies (case control or cohort) aimed at evaluating the association between malaria (stratified by different manifestations of malaria) and NTS bacteraemia, should include suitable controls from the communities, in order to confirm this association. At the same time, there is a pressing need for improved point of care diagnostics for severe bacterial infection across sub-Saharan Africa, both for patients without malaria and those whose malaria infection is complicated by severe bacterial infection. Intervention studies (trials), focused on reducing the burden of malaria which include NTS bacteraemia as an endpoint, will help to corroborate the causal relationship. A reduction in the burden of malaria is likely to simultaneously reduce the burden of NTS bacteraemia and should be a priority.
